# Biomedical Grade Stainless Steel Coating of Polycaffeic Acid via Combined Oxidative and Ultraviolet Light-Assisted Polymerization Process for Bioactive Implant Application

**DOI:** 10.3390/polym11040584

**Published:** 2019-04-01

**Authors:** Ludwig Erik Aguilar, Ji Yeon Lee, Chan Hee Park, Cheol Sang Kim

**Affiliations:** 1 Department of Bionanosystem Engineering, Chonbuk National University, Jeonju City 54001, Korea; leaguilar@jbnu.ac.kr; 2 Department of Mechanical Design Engineering, Graduate School, Chonbuk National University, Jeonju City 54001, Korea; swc2736630@gmail.com; 3 Division of Mechanical Design Engineering, Chonbuk National University, Jeonju City 54001, Korea

**Keywords:** stainless steel, surface modification, wettability, polycaffeic acid, biocompatibility

## Abstract

Stainless steel as a biomedical implant material has been studied in various fields and in various forms, such as vascular stents, bone plates, dental screws, and artificial hip and bone material. In this study, we used polycaffeic acid (PCA), a natural phenolic compound, to coat the surface of medical grade stainless steel to provide added potential medicinal effects by virtue of its inherent anti-inflammatory, antiviral, antifibrosis, antithrombosis, and antihypertensive characteristics. We did this via UV irradiation under an alkaline state to solve the cost and time problems of other existing coating methods. The physicochemical properties of the samples were investigated through field emission scanning electron microscopy (FESEM), atomic force microscopy (AFM), contact angle, FTIR, and X-ray photoelectron spectroscopy (XPS). Surface bioactivity using NIH-3T3 cell lines were observed in vitro. We expect that the proposed methodology may contribute to the field of study of implantable metallic devices.

## 1. Introduction

The use of metallic implants has been a standard in the medical field in augmenting, restoring, and treating various physiological impediments [1,2,3]. Various forms of metallic devices such as stainless steel, titanium, chromium cobalt, and nickel titanium alloys have been developed, which goes hand in hand with the development of the surface treatment of these aforementioned devices [4,5,6]. Metallic implants ideally should not cause any adverse effects in the biological systems they are applied to; however, due to improper handling and unforeseen physiological responses, side effects may arise that can limit the effectivity of these metallic implants. 

There are various surface treatments that are standard in fabricating metallic implants. These treatments vary from galvanization to the promotion of corrosion resistance to the addition of passive oxide layers on the surfaces. However, these surface modifications are intended to increase the inertness of these implants, and recent trends have been pointing to the addition of bioactive molecules to incorporate therapeutic effects and increase the cellular response to implantable materials [2,5]. Stainless steel (SS) has special characteristics as an alloy that can be used inside the body due to its high corrosion resistance and biological compliance. In order to improve devices composed of stainless steel, bioactive molecules can be added to the surface of SS substrates via adhesion and covalent bonding processes. Examples of this are in the use of polydopamine, hyaluronic acid, heparin, fucoidan, chondroitin sulfate, and antioxidant compounds, as well as extracellular matrix (ECM) proteins for functionalization, to improve cell–material interactions that can promote endothelial cell formation, osteointegration, and differentiation [7,8,9].

Recently, materials containing polyphenols extracted from natural sources with various biological effects have been emerging as implant coatings. In particular, polyphenols extracted from various kinds of tea have been studied as anticancer and cardiovascular protective compounds. Polyphenol compounds contained in green tea, black tea, and even coffee have beneficial effects such as anti-proliferative, anti-inflammatory, antioxidant, anti-thrombogenic, and endothelial activities [10]. Among the various phenolic compounds such as caffeic acid (CA), pyrogallol (PG), gallic acid (GA), and tannic acid (TA), CA is considered to be a novel therapeutic strategy for various diseases because of its antioxidant, anti-thrombosis, antibacterial, anti-inflammatory, antiviral, and antihypertensive properties that have been demonstrated in recent studies [11]. Using this knowledge, we can alleviate the multiple side effects of metallic implants with the use of polycaffeic acid. Additionally, we can make sure that the innate biomedical properties of this phenolic compound will take effect. It can potentially be applied to vascular stents to reduce the inflammatory response to endothelial cells, and reduce the risk of thrombus formation in the presence of whole blood [12]. Other metallic implants such as bone screws and plates can also be improved by employing the antibacterial property of caffeic acid, which reduces biofilm formation and therefore minimizes the risk of implant failure and replacement, thus greatly improving the quality of life of the patient [13]. 

In addition, the process of UV irradiation can accelerate oxidative polymerization and the deposition of polycaffeic acid (PCA). This is a new method that incorporates light in order to trigger the polymerization process of phenolics and avoid the use of highly oxidative agents. This will help in avoiding contaminants and residue that could affect the biocompatibility of the implant material. The use of a UV-induced polymerization process in coating polycaffeic acid on the 316L SS substrate can be used to extend the area of bioactive materials.

## 2. Materials and Method

### 2.1. Materials

A SS316L sheet (0.3 mm thick) was purchased from Shandong Xingya Tesico Co. The sheet was cut into small pieces, each with the dimensions of 2.5 cm × 5 cm. Caffeic acid (CA, 3,4-dihydroxybenzeneacrylic acid, MW = 180.16 g/mol) and sodium carbonate (Na_2_CO_3_, MW = 105.99 g/mol) was purchased from Sigma Chemical Co. (Daejon, South Korea) and used without further purification. Each chemical product such as sodium bicarbonate (NaHCO_3_, MW = 84.01 g/mol), potassium chloride (KCl, MW = 74.55 g/mol), hydrofluoric acid (HF, MW = 20.0063 g/mol), and nitric acid were all purchased from DC Chemical Co. (Shanghai, China) and Kanto Chemical Co. (Seoul, Korea) All aqueous solutions (buffer systems) were prepared using ultrapure water purified with a Milli-Q UV-Plus water purification system (Bedford Millipore, Darmstadt, Germany). The water had a resistivity of >1018 MΩ·cm^−1^.

### 2.2. Preparation of Samples

**Figure 1:**
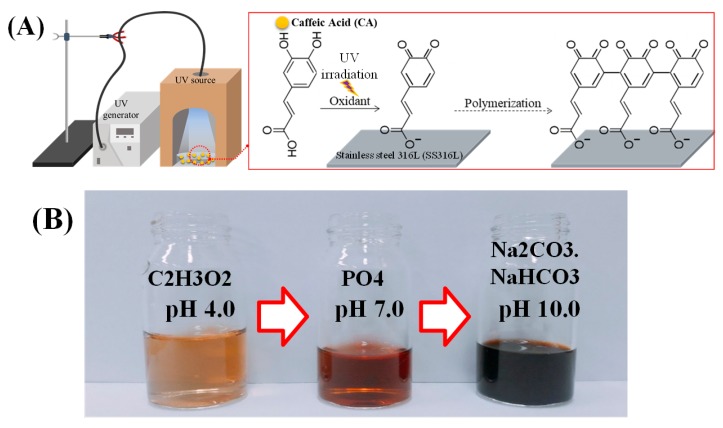
(**A**) Schematic illustration of the experimental setup for caffeic acid polymerization via oxidative and UV irradiation processes on a stainless steel 316L substrate and the proposed mechanism for the polymerization of caffeic acid. (**B**) Photographs of the caffeic acid solution after UV irradiation at different pH levels in a carbonate-bicarbonate buffer solution.

Each of the SS316L pieces were prepared by the following chemical etching method. SS316L pieces were submerged into a solution of hydrofluoric acid (HF, 7 wt%), then nitric acid (HNO_3_, 12%), and lastly, distilled water (DW, 81 wt%) for about 20 s each. This was followed by instant sonication, first in ethanol, and then in DW for 10 min each. The etched SS316L pieces were then dried in a nitrogen atmosphere to avoid oxide layer formation. The prepared etched-SS316L pieces were then soaked in the CA solution (0.5 mM of CA powder in 30 mL of 0.1 mM carbonate-bicarbonate buffer solution, pH 10) and UV irradiated at a 95% power setting using an Omnicure Series 1500 lamp for 4 h with the distance between the top of the solution and the tip where light is emitted being 3 cm, and with slow stirring in room temperature, as shown in Figure 1. The samples were then named as follows: untreated SS316L (the control), Etched-SS316L, and PCA-SS316L.

### 2.3. Characterization of Surface Properties

The surface morphology of the prepared samples was surveyed using field emission scanning electron microscopy (FESEM, Carl Zeiss Supra 40VP, Oberkochen, Germany) with an energy dispersive X-ray spectroscopy (EDS, Carl Zeiss Supra 40VP, Oberkochen, Germany) system to analyze the elemental composition of the specimens’ surface (elemental mapping). The samples were sputter-coated under argon in order to make them electrically conductive. The excitation voltage used to capture the images was set at 5 kV. In order to analyze the degree of surface roughness of the prepared samples, atomic force microscopy (AFM, Bruker Multimode-8, Bruker, MA, USA) was conducted. The internal bonding architecture of the samples was checked using Fourier transform infrared spectroscopy (FTIR, Perkin Elmer Co., Waltham, MA, USA) with a scanning range of 400–4000 cm^−1^, and X-ray photoelectron spectroscopy (XPS, AXIS-NOVA, Kratos, Inc., Manchester, UK) with an Al Kα irradiation source. The hydrophilicity and hydrophobicity of samples were measured using a contact angle meter (GBX, Digidrop, Dublin, Ireland) using deionized water.

### 2.4. UV-VIS Spectroscopy Study on the Effect of pH and Presence of UV Irradiation in Caffeic Acid Polymerization

CA (0.05 mM) was dissolved in different buffer solutions (pH 4.0 acetate buffer, pH 7.0 phosphate buffer, and pH 10.0 sodium carbonate-bicarbonate buffer). The CA solutions were then UV irradiated for (5 min Supplemental Figure S2) and (4 h Supplemental Figure S1) separately to assess the effect of UV light in the degree of polymerization of CA. We compared this to the CA solutions under dark conditions (buffer-only and covered to create conditions in the absence of light). The resulting CA solutions were subsequently analyzed using UV-VIS spectroscopy (SCINCO MEGA 800, Seoul, South Korea) at 200–800 nm wavelengths. 

### 2.5. In Vitro Bioactivity Studies on Different SS316 Substrates

For the bioactivity evaluation, we used fibroblast cells (NIH-3T3) to characterize the biocompatibility of each sample. Prior to seeding, NIH-3T3 cells were cultured in Dulbecco’s modified Eagle’s medium (DMEM, Sigma Chemical Co., Daejon, South Korea) with 10% fetal bovine serum (FBS, Sigma Chemical Co.) and 1% penicillin/streptomycin. Different samples (untreated SS316L, Etched-SS316L, and PCA-SS316L) were cut to their proper size to fit into the 96-well cell culture plate (SPL Life Science, Pocheon, Korea). The samples were sterilized with UV irradiation and washed three times with 1% phosphate buffered saline (PBS). The NIH-3T3 cells were seeded onto the samples at a density of 2 × 10^3^ cells/well. Cell biocompatibility was evaluated at specific time points of 1, 3 and 5 days by adding 10 µL of Dojindo CCK-8 solution to each well. The samples were incubated for 2 h in a 37 °C incubator with 5% CO_2_, and the optical density (OD) of the media of each sample was measured at 450 nm using a microplate reader (Tecan, Männedorf, Switzerland). The data presented are the average results of triplicated experiments.

The cell morphology and distribution of NIH-3T3 cells on each sample (untreated SS316L, Etched-SS316L, and PCA-SS316L) were evaluated after 5 days using a super-resolution confocal laser scanning microscope (SR CLSM, LSM 880, Carl Zeiss, Oberkochen, Germany). Beforehand, the NIH-3T3 cells on samples were fixed in 4% paraformaldehyde solution overnight at 4 °C and then washed three times with 1% PBS. After this, the NIH-3T3 cells were immersed in 0.5% Triton X-100 for 2 min at room temperature and then washed twice with 1% PBS. After blocking with 1% human serum albumin (HSA)/PBS dilution for 30 min, the cells in each of the samples were stained with ActinGreen and DAPI for actin filament staining and cell nuclei visualization, respectively. 

### 2.6. Statistics

All data were independently repeated at least three times, and data are presented as the mean ± standard deviation (SD), with images analyzed by ImageJ software. A one-way ANOVA and Student’s *t*-test were used to compare the data obtained from different samples under identical experimental conditions. A *p*-value of less than 0.05 was considered statistically significant.

## 3. Results and Discussion

Figure 1A shows the setup and polymerization process of caffeic acid onto an etched stainless steel 316L sample. Figure 1A indicates the caffeic acid, in the state of phenolic monomer, undergoing UV irradiation in the solution. This leads to rapid ionization, resulting in adhesion on the etched stainless steel, and a polymerization phenomenon in which the caffeic acid monomers aggregate to form a polymeric state. 

Prior to the polymerization of caffeic acid through UV irradiation, the experiment as follows was conducted to confirm the degree of polymerization according to the pH of the buffer solution. The pH levels of the different buffer solutions were determined to be 4.0 (acetate acidic condition), 7.0 (phosphate neutral), and 10.0 (sodium carbonate bicarbonate basic condition). As shown in Figure 1B, the color of the buffer solution changed from yellow to brown to dark brown depending on the pH of the buffer, with pH 10 showing the darkest color change. The caffeic acid in the different pH buffer conditions also showed the shifting of the absorbance wavelength from the UV range to the visible region (Supplemental Figure S2). We intended to have a high degree of polymerization as much as possible, and it was evident that the Na_2_CO_3_–NaHCO_3_ buffer solution (pH 10) promotes the highest degree of polymerization. Therefore, all the following experiments were carried out with a buffer solution at pH 10.0. We also proved that in using an alkali condition alone, the polymerization process is not enough to induce a coating on the SS substrate. The polymerization of CA under the UV condition is much higher, as evidenced by the shifting of the UV absorbance to the visible spectrum (Supplemental Figure S1). The alkali condition helps the ionization of caffeic acid under UV irradiation, leading to a much higher degree of phenolic polymerization [14]. The UV light can accelerate the polymerization process due to its ability to form reactive oxygen species (ROS), singlet oxygen (1O2), superoxide radicals (O2−•), or highly reactive hydroxyl radicals (•OH), even in the presence of trace oxygen species. ROS formation is important in the polymerization process because it activates quinone formation in the catechol moiety of plant phenolics. 

**Figure 2:**
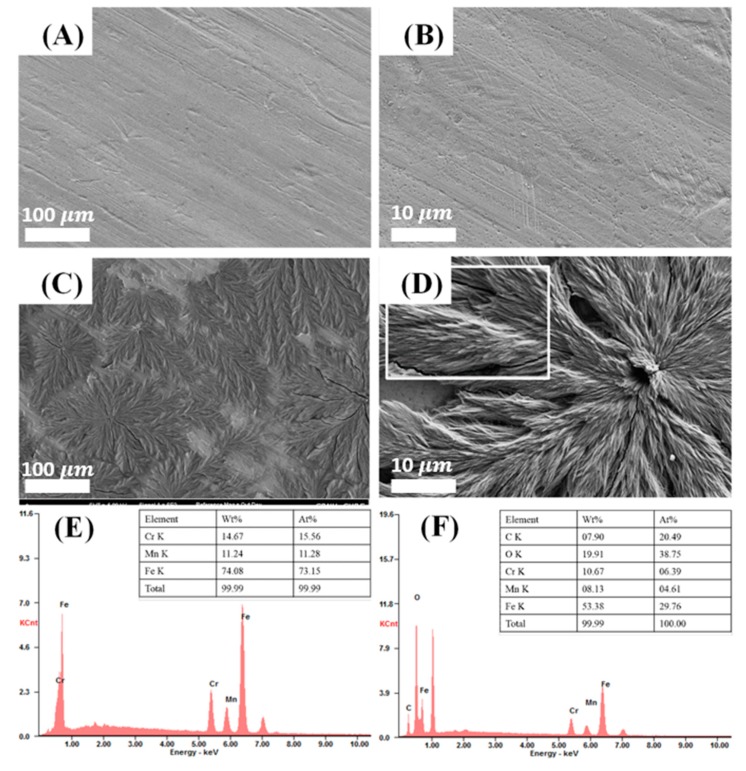
(**A**–**D**) Field emission scanning electron microscopy (FESEM) images in two magnitudes (×1000, 5000) of stainless steel substrates with/without polycaffeic acid coating. Energy dispersive X-ray spectroscopy (EDS) spectra showing the main components present on the surface of (**E**) etched stainless steel 316L and (**F**) stainless steel 316L coated with polycaffeic acid.

Figure 2A–D show the surface of each substrate through FESEM with Figure 2A,B showing the surface of the stainless steel 316L after the chemical etching process (Etched-SS316L), and with Figure 2C,D showing the surface of the PCA-coated specimen on Etched-SS316L (PCA-SS316L). The surface of Etched-SS316L was found to be smooth in appearance, except for some scratches due to the chemical etching process. In PCA-SS316L, we could observe several leaf-like shapes appearing to grow on the surface of the substrate due to the coating of PCA, with the enlarged photograph shown in Figure 2D demonstrating that PCA is formed by aggregation. Figure 2E,F show the survey of the main elements on the surface of each substrate, and the table located in the graph shows the exact percentage of each element. According to the table, Etched-SS316L is composed of Cr, Mn, and Fe at 15.56, 11.28, and 73.15 at%, respectively, and it lacks the presence of organic elements such as oxygen and carbon. However, PCA-SS316L has the presence of organic material and is composed of C, O, Cr, Mn, and Fe at 20.49, 38.75, 06.39, 04.61, and 29.76 at%, respectively.

**Figure 3:**
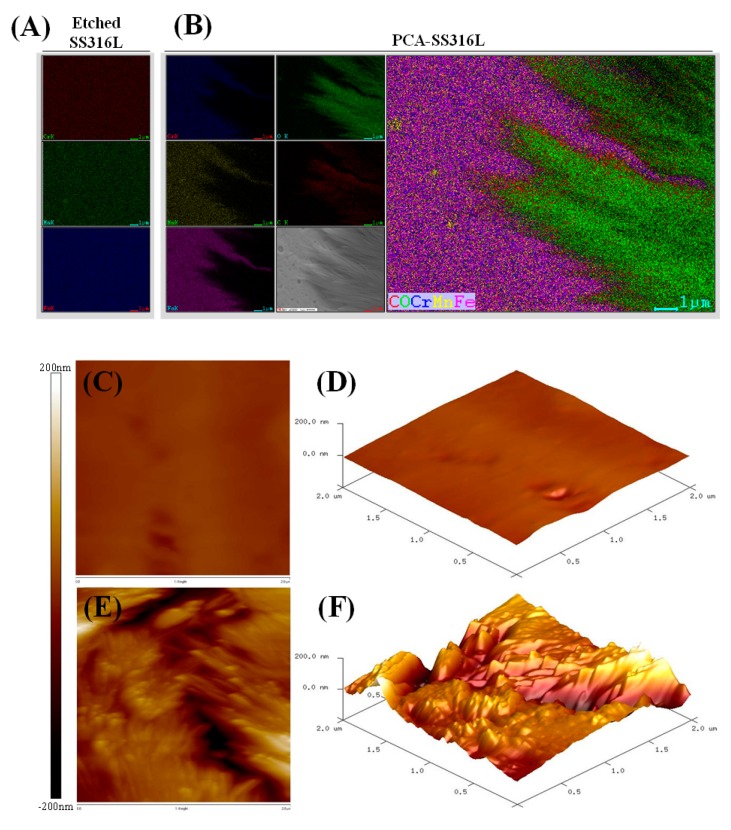
Surface analysis via (**A**,**B**) EDS mapping of the elemental species on the surface of etched stainless steel 316L and stainless steel 316L coated with polycaffeic acid. Two-dimensional (2D) and three-dimensional (3D) surface atomic force microscopy (AFM) images (2 µm × 2 µm); (**C**,**D**) etched stainless steel 316L foil; and (**E**,**F**) stainless steel 316L foil coated with polycaffeic acid.

To visually survey the elemental distribution on the surface of the different SS316L samples, EDS mapping was conducted. Figure 3A,B show the mapping results of Etched-SS316L and PCA-SS316L. The mapping demonstrates the presence of organic material on top of the PCA-SS316L sample. Carbon and oxygen were detected exactly in the area where the leaf-like structures were located. Caffeic acid is composed of only three elements (carbon, oxygen, and hydrogen). Also, the surface topology further corroborates the difference in the surface characteristics of the two SS316L samples as shown in Figure 3C–F. Both two-dimensional (2D) and three dimensional (3D) images show that, unlike the Etched SS316L which had a smooth surface, PCA-SS316L had a rough surface with 200 nm variations due to the coating of PCA. The surface analysis proved that there is an organic coating on the surface of the PCA-SS316L. The enhanced surface roughness can affect other properties that are relevant to biomedical applications. Since the surfaces of untreated SS316L and Etched-SS316L are smoother in comparison to that of PCA-SS316L, the cellular activity on the surface on PCA-SS316L can be enhanced. Increased surface roughness plays a role in cellular activity due to the increased surface area needed for cell attachment [15]. 

**Figure 4:**
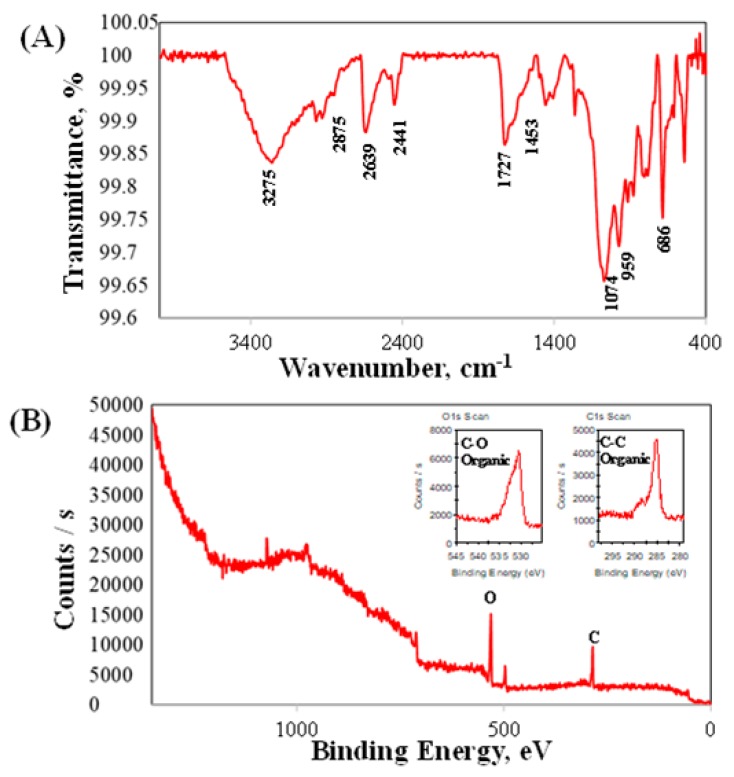
Analysis of chemical composition for the polycaffeic acid coatings: (**A**) FTIR spectra; (**B**) survey spectra of X-ray photoelectron spectroscopy (XPS). The XPS high-resolution O1s and C1s spectra of the stainless steel 316L covered by polycaffeic acid are located on the graph (**B**).

Physicochemical analysis for the presence of polycaffeic acid was proven using the following FTIR and XPS data. The surface chemical analysis on the PCA-SS316L sample indicated the following IR absorption peaks. In Figure 4A, we can attribute the 3275, 2639 cm^−1^ peaks to OH functional groups with H-bonds, 2875 cm^−1^ to the =C–H stretch, 1727 cm^−1^ to the C=O stretch, and 1074 cm^−1^ to the C–O ester groups. These functional groups are similar to what was observed in the caffeic acid molecule with slight peak shifts occurring due to the polymerization of CA during the coating process. To corroborate the FTIR results and determine the presence of an organic coating on the SS substrate, we analyzed the PCA-SS316L sample with X-ray photon spectroscopy (Figure 4B). The C1s and O1s scans indicated the presence of organic oxygen with C–O bonding at 531 eV. The C1s scan was also indicative of C–C bonding at 284 eV. This is evidence of organic compounds present on the surface of the PCA-SS316L sample. Pure metallic substrates will not exhibit these organic peaks due to passive oxide layers being present in metallic alloys once exposed to the atmosphere. However, with the presence of the PCA coating, these functional groups were detected.

**Figure 5:**
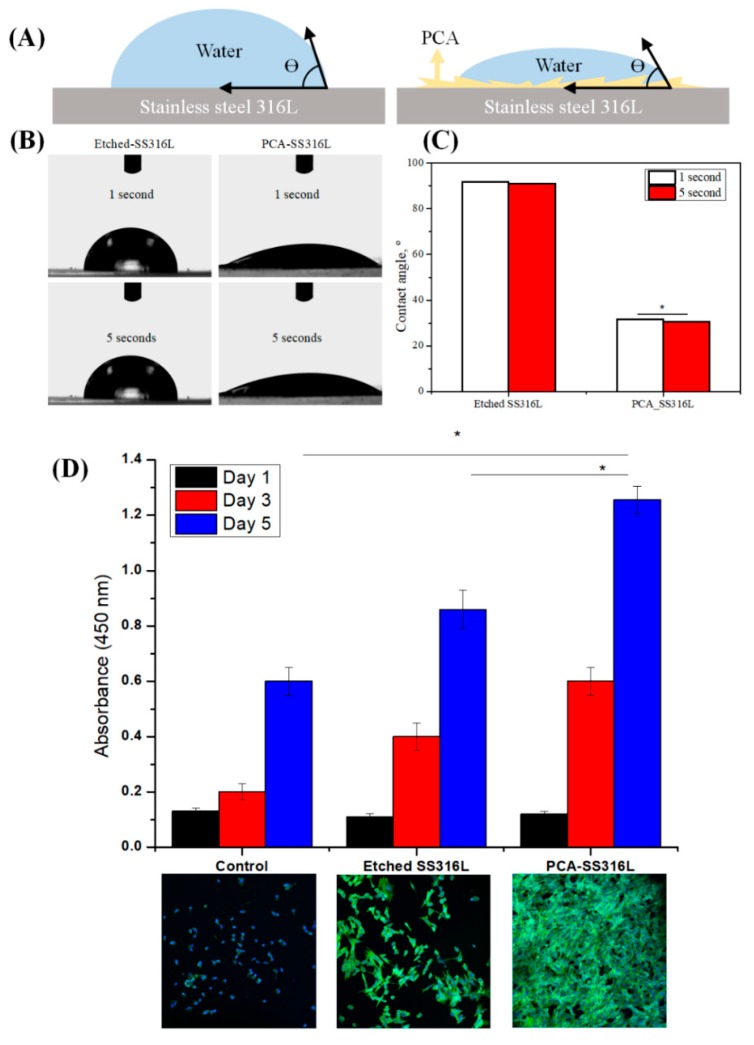
(**A**) Schematic showing a water droplet sitting on an etched stainless steel 316L substrate and a stainless steel 316L substrate covered by polycaffeic acid. (**B**) Optical images showing a macroscopic water droplet on an etched stainless steel 316L substrate and a stainless steel 316L substrate covered by polycaffeic acid at 1 and 5 s. (**C**) Comparison of the contact angle value of each substrate at 1 and 5 s, respectively. (**D**) NIH-3T3 cell proliferation on the control etched stainless steel 316L and stainless steel 316L covered with polycaffeic acid for 1, 3, and 5 days as measured by a CCK-8 assay. Inset: Confocal images showing the morphology of NIH-3T3 cells of substrates on different coatings after 5 days. Data are presented as the mean ± SD (n = 3).

To assess another physical property that is relevant to biotechnology, we subjected the Etched-SS316L and PCA-SS316L samples to water contact angle measurements. As seen in Figure 5A–C, the contact angle measurements for the Etched-SS316L and PCA-SS316L were statistically significant at both time points. The water droplet readily diffuses on the surface of the PCA-SS316L sample with a mean angle of 35° compared to the Etched-SS316L sample at 90°. Its hydrophilic property was enhanced due to the increased polarity in the surface of the PCA-SS316L sample. The presence of the –OH groups in the PCA coating created an electronegative polar surface that would be beneficial when we want to increase the adhesion of cellular proteins such as cell adhesion molecules (CAM) [16]. 

The biocompatibility of the PCA coating was determined via CCK-8 cell counting assay (Figure 5D). The amount of actively metabolic cells on the surface of both Etched-SS316L and PCA-SS316L substrates were observed, and the results show a statistically significant increase compared with the control substrate (untreated SS316L). The increased wettability and surface area can be attributed to this result. Cell adhesion molecules (CAM) can be readily adsorbed on the surface of the treated surfaces, therefore increasing the attachment, proliferation, and compatibility of the NIH-3T3 fibroblast cells [17]. To further corroborate the CCK-8 assay, immunostaining was conducted with DAPI and ActinGreen. Here we can see the difference with the Etched-SS316L and PCA-SS316L samples. The morphological features of the fibroblasts were different in both samples, and it can be seen in the PCA-SS316L (Figure 5D inset image) that the cytoskeleton of the cells was more spread out compared with that of the cells in the Etched-SS316L and untreated SS316L. 

We found that polycaffeic acid could be applied to the surface of stainless steel 316L, which is the most typical implant material used inside the body. It is beneficial as an implantable material, not only to exhibit biocompatibility, but also to have added bioactive properties, which is why we probed the possibility of using a therapeutic phenolic as a coating material. Polycaffeic acid can enhance the bioactivity of the SS implant by creating an antibacterial, anti-inflammatory, and anti-viral interface. The coating was characterized and compared in activity to etched SS316L samples, and it was found that the coated sample had higher wettability, surface roughness, and bioactivity compared with the control groups. 

The characterization (FESEM, EDS, AFM, FT-IR, XPS) data verify that the coating technique is effective in creating a functional layer on top of an SS substrate. SS does not have inherent biofunctional properties such as blood compatibility, osteoconductivity, and bacterial resistance. Adding active molecules is one way of making the implant material bioactive. Polyphenols are great candidates in making implantable materials active due to their inherent medicinal properties. Caffeic acid in particular has been proven to be a potential agent in the reduction of bacterial colonization, inflammation, and oxidation to biological tissues [14]. 

## 4. Conclusions

The data we present here tell us that using PCA as a coating material can enhance the wettability and surface area of the metal substrate, therefore creating a more bioactive surface. This could have potential applications for materials such as metallic bone implants and vascular stenting. Problems in these areas involve increased nosocomial infection due to poor handling of materials, as well as restenosis that arises due to acute inflammatory response. PCA could avoid these scenarios by creating a functional layer interface between the biological tissue of importance and the implant surface.

## Supplementary Materials

The following are available online at https://www.mdpi.com/2073-4360/11/4/584/s1: Figure S1. Caffeic acid UV-VIS spectra in two different conditions—(DC) dark condition and (UV) UV irradiated condition. Inset image shows actual polycaffeic solution sodium carbonate bicarbonate buffer, pH 10; Figure S2. Caffeic acid UV-VIS spectra in two different conditions—(DC) dark condition and (UV) UV irradiated condition (5 min exposure) and different pH buffer media (pH 4.0 acetate, pH 7.0 phosphate, pH 10.0 sodium carbonate-bicarbonate).
