# Supplementary material for: Biomedical Grade Stainless Steel Coating of Polycaffeic Acid via Combined Oxidative and Ultraviolet Light-Assisted Polymerization Process for Bioactive Implant Application
Source: Polymers (Basel). 2019 Apr 1;11(4):584. doi: 10.3390/polym11040584 (PMC6523249; doi:10.3390/polym11040584)
Supplement: Supplementary file 1 [file polymers-11-00584-s001.pdf]

Supplementary Information

Biomedical Grade Stainless Steel Coating of Polycaffeic Acid via Combined Oxidative and Ultra Violet Light Assisted Polymerization Process for Bioactive Implant Application

Ludwig Erik Aguilar[1,+], Ji Yeon Lee[2,+], Chan Hee Park[2,3,*], Cheol Sang Kim[2,3,*]

[1]*Department of Bionanosystem Engineering, Chonbuk National University, Jeonju City, Republic of Korea*

[2]*Department of Mechanical Design Engineering, Graduate School, Chonbuk National University, Jeonju City, Republic of Korea*

[3]*Division of Mechanical Design Engineering, Chonbuk National Universiry, Jeonju City, Republic of Korea*

Correspondence: biochan@jbnu.ac.kr, chskim@jbnu.ac.kr

[+] Equally Contributed Authors

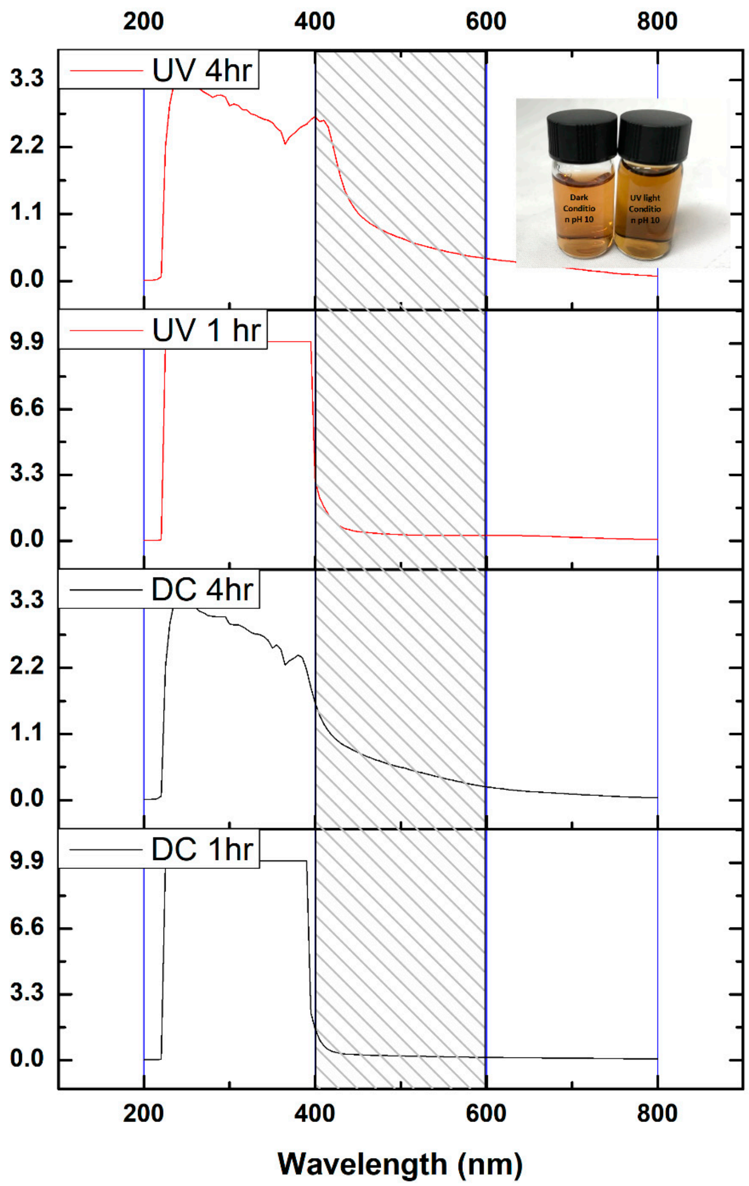

Figure S1. **Caffeic acid UV-Vis spectra in two different conditions (DC) dark condition and (UV) uv irradiated condition. (inset image) Actual polycaffeic solution sodium carbonate bicarbonate buffer pH 10.**

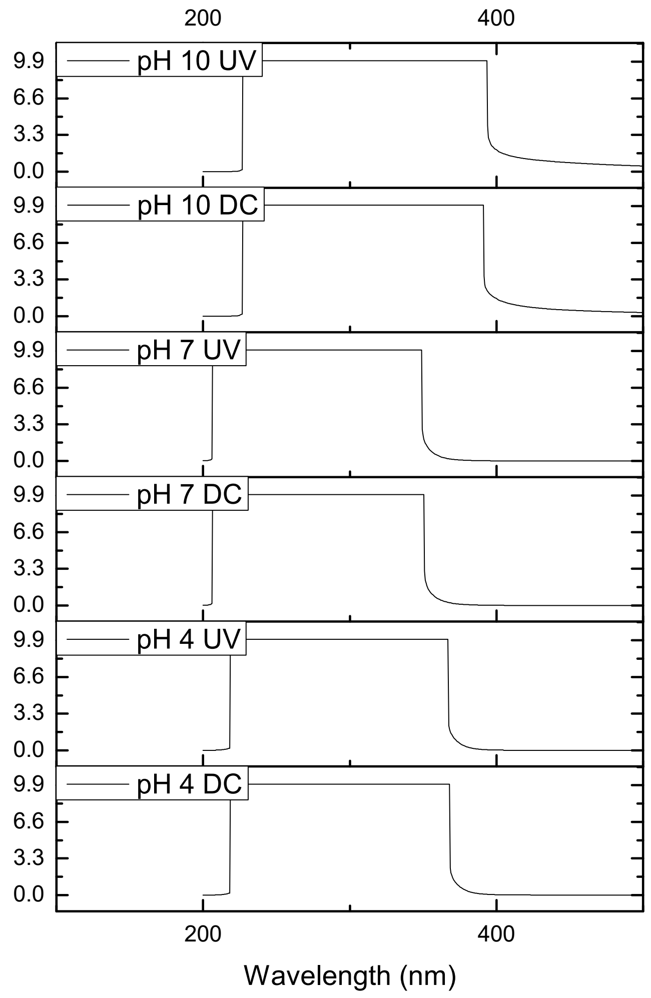

Figure S2. **Caffeic acid UV-Vis spectra in two different conditions (DC) dark condition and (UV) uv irradiated condition (5mins exposure) and different pH buffer media. (ph 4.0 acetate, pH 7.0 phosphate, pH 10.0 Sodium Carbonate Bicarbonate).**
